# Efficacy of Intraoperative Implant Prophylaxis in Reducing Intraoperative Microbial Contamination

**DOI:** 10.1177/2192568218780676

**Published:** 2018-06-12

**Authors:** Aakash Agarwal, Boren Lin, Jeffrey C. Wang, Christian Schultz, Steve R. Garfin, Vijay K. Goel, Neel Anand, Anand K. Agarwal

**Affiliations:** 1 University of Toledo, Toledo, OH, USA; 2 USC Spine Center, Los Angeles, CA, USA; 3 Apex Spine Center, München, Germany; 4 University of California, San Diego, CA, USA; 5 Cedars Sinai Medical Center, Los Angeles, CA, USA

**Keywords:** surgical site infection, infection, asepsis, bioburden, pedicle screw, spine surgery, cross-contamination, microbes

## Abstract

**Study Design:**

A prospective single-center study.

**Objectives:**

Assess to what degree contamination of pedicle screws occur in standard intraoperative practice and if use of an impermeable guard could mitigate or reduce such an occurrence.

**Methods:**

Two groups of sterile prepackaged pedicle screws, one with an intraoperative guard (group 1) and the other without such a guard (group 2), each consisting of 5 samples distributed over 3 time points, were loaded onto the insertion device by the scrub tech and left on the sterile table. Approximately 20 minutes later, the lead surgeon who had just finished preparing the surgical site touches the pedicle screw. Then instead of implantation it was transferred to a sterile container using fresh clean gloves for bacterial and gene analysis. Guarded screw implies that even after unwrapping from the package, the screw carries an impermeable barrier along its entire length, which is only removed seconds prior to implantation.

**Results:**

The standard unguarded pedicle screws presented bioburden in the range of 10^5^ to 10^7^ (colony forming units/implant) with bacterial genus mostly consisting of *Staphylococcus* and *Micrococcus*, the 2 most common genera found in surgical site infection reports. The common species among them were *Staphylococcus epidermis*, *Staphylococcus aureus*, *Micrococcus luteus*, and *Staphylococcus pettenkoferi*, whereas the guarded pedicle screws showed no bioburden.

**Conclusions:**

Shielding the pedicle screws intraoperatively using a guard provides a superior level of asepsis than currently practiced. All unshielded pedicles screws were carrying bioburden of virulent bacterial species, which provides an opportunity for the development of postoperative infections.

## Introduction

Spinal infections continue to be a significant problem both clinically for patients and socioeconomically in terms of growing health care costs. The average hospitalization cost resulting from such infections is $63 000 per case at an average frequency of 5.5% of cases (built in cost of $3465 for every surgery).^1,2^ Consequently, in recent years, there has been considerable interest in refining aseptic techniques, such as intraoperative handling of implantable devices, in order to reduce the bioburden being transmitted to the patients, a majority of whom are also immunocompromised. Researchers have already detected colony-forming units (CFUs) of bacteria on exposed sterile implants and the gloves from the surgeon, scrub nurse, and assistants. Furthermore, they have also found significant reduction in surgical site infection (SSI) rates in spine surgery due to stand-alone measures, like glove change before touching the implants.^3-13^ In addition, recent literature is growing with studies that look at process variables in the operating room (OR), for example, keeping implants covered until the immediate time of use, reducing OR traffic, avoiding reprocessing of implants (ie, providing sterile prepackaged single-use implants), and to avoid touching the implants altogether. Last, contemporary fields like plastic surgery and general surgery have already adapted such practices, through the use of Keller funnels, and wound protectors, respectively, which prevent cross-contamination between the wound, gloves, and the implants (irrigation fluid for the latter).^12-15^ The purpose of this study was 2-fold, to evaluate the bioburden and the species of bacteria present on each pedicle screw being implanted, and the efficacy of an intraoperative guard in reducing such occurrences.

## Method

**Figure 1.**
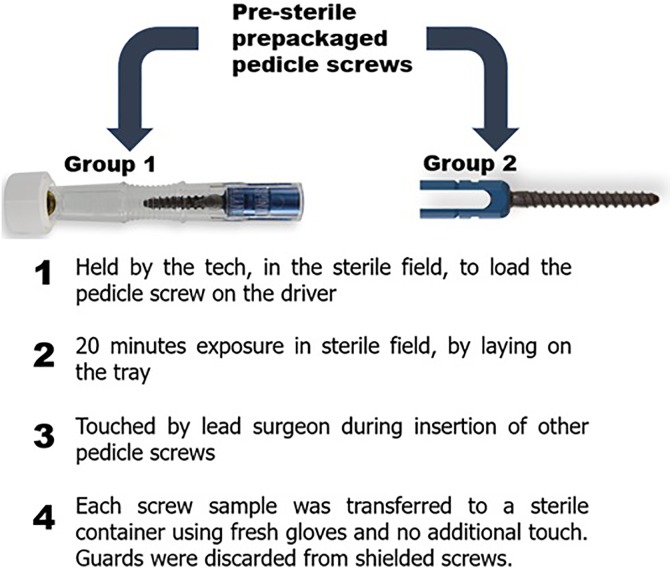
Schematic showing the intraoperative study design for consistency among the 2 groups.

No institutional review board approval was needed for this study. The study consisted of 2 groups of sterile prepackaged pedicle screws, one with an intraoperative guard (group 1) and the other without such a guard (group 2). Each group consisted of 5 samples distributed over 3 time points (spinal fusion surgeries), and each was performed in a different OR, with a different surgeon and surgical staffs (Table 1). During surgery, each of the test screws were loaded by the scrub tech and were left on the sterile table. Approximately 20 minutes later, the lead surgeon who had just finished preparing the surgical site touches the pedicle screw (Figure 1). Then, instead of implantation, it was transferred to a sterile container using fresh clean gloves for bacterial analysis. This 3-step protocol was conceived through consensus among more than 50 orthopedic spine surgeons and neurosurgeons across the globe. The time period of 20 minutes was the shortest time of exposure that was communicated to us. The guards from the guarded group (group 1) of pedicle screws were discarded before the transfer. Inclusion criteria for each surgery included 1- to 2-level spinal fusion for degenerative or traumatic spine pathology with patients aged ≥18 years. Furthermore, all standards of care were kept unaltered. The collected implant was transferred to 25 mL of tryptic soy broth (TSB; Sigma-Aldrich, St. Louis, MO) within a time period of 4 hours. The tube with the sample was filled with broth and then put inside a 50 mL conical centrifuge tube. This was followed by incubation in a rotisserie incubator (Fisher Scientific, Hampton, NH) at 36°C and 12 revolutions per minute. After 24 hours of incubation, the turbidity of extracts from each sample was measured by spectrophotometry at 600 nm. In addition, triplicates of 0.1 mL from each sample were streaked on individual Columbia agar plate with 5% sheep blood (Carolina, Burlington, NC) and incubated at 36°C. The incubation of the remaining volume was continued in an incubation shaker (Fisher Scientific), using a different flash at 36°C and 200 revolutions per minute. After completion, the extracts from each sample (from both the groups) were send for 16sRNA gene sequencing for bacterial identification.

**Table 1.** The Sample Size and Its Distribution Over 3 Time Points (Surgeries), Surgeons, Scrub Tech, and Operating Room.

Sample Number	Group	Surgery	Operating Room	Scrub Tech	Surgeon	Screw Type
1	1	A	A	A	A	5.5 × 50 mm
1	2					5.5 × 50 mm
2	1					7.5 × 60 mm
2	2					7.5 × 60 mm
3	1	B	B	B	B	7.5 × 60 mm
3	2					7.5 × 60 mm
4	1	C	C	C	C	7.5 × 60 mm
4	2					7.5 × 60 mm
5	1					7.5 × 60 mm
5	2					7.5 × 60 mm

## Results

**Figure 2.**
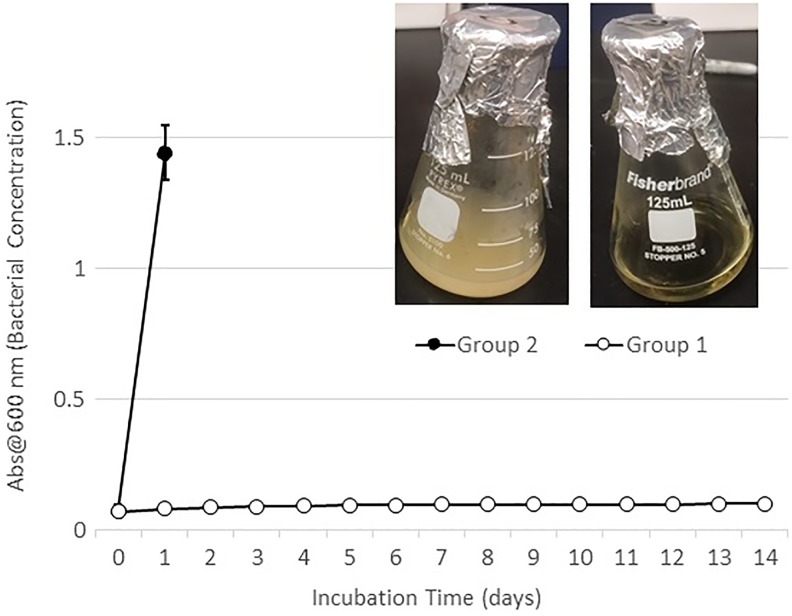
Spectroscopy data showing saturated levels of growth within 24 hours in group 2, versus no growth for 14 days in group 1.

**Figure 3.**
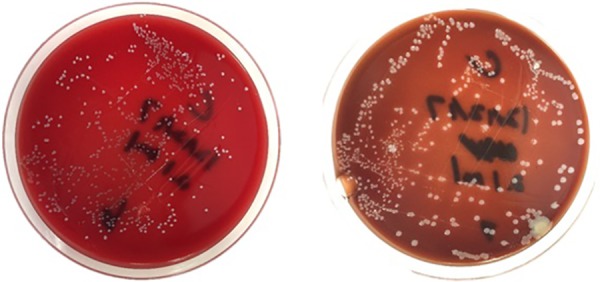
Colony-forming units on sample 1 of group 2, at 24 hours (left) and 4 days (right) after streaking. These represent 2 of 0.1 mL triplicates that were cultured on plates.

**Figure 4. fig4-2192568218780676:**
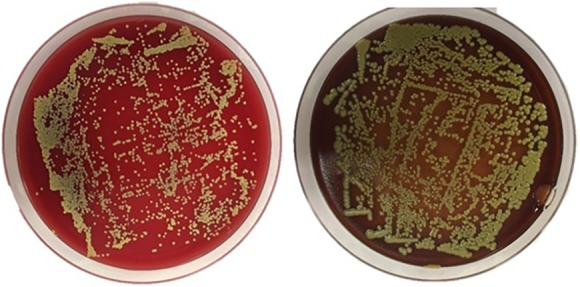
Colony-forming units on sample 2 of group 2, at 24 hours (left) after streaking. These represent 2 of 0.1 mL triplicates that were cultured on plates.

**Figure 5. fig5-2192568218780676:**
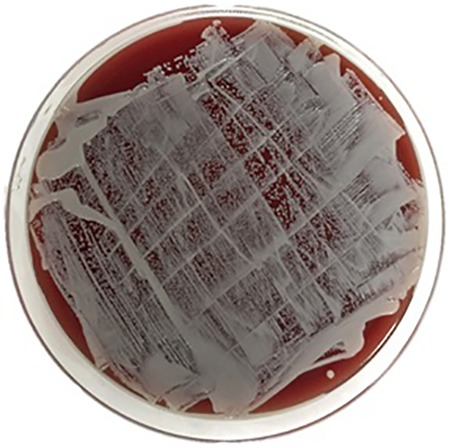
Colony-forming units on sample 3 of group 2, at 24 hours after streaking. They reached confluency at 24 hours and were not incubated any further. This represents one of 0.1 mL triplicates that were cultured on plates.

**Figure 6. fig6-2192568218780676:**
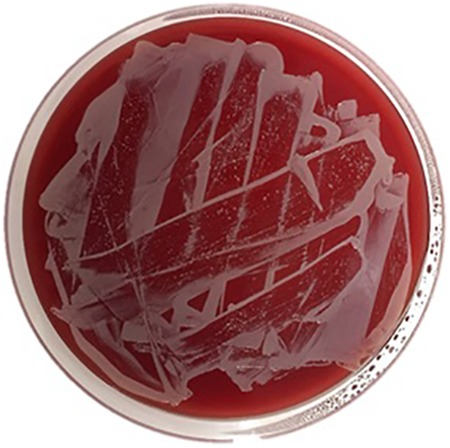
Colony-forming units on sample 2 of group 2, at 24 hours after streaking. They reached confluency at 24 hours and were not incubated any further. This represents one of 0.1 mL triplicates that were cultured on plates.

Spectrophotometry results detected saturated levels of turbidity within 24 hours in samples from group 2 (Figure 2). The samples from group 1 showed no turbidity for the entire duration of the incubation period (14 days; Figure 2). Every plate from each sample of group 2 had visible CFUs growth within 24 hours past streaking (Figures 3 [Fig fig4-2192568218780676] [Fig fig5-2192568218780676] [Fig fig6-2192568218780676]–7). The total CFUs ranged from 10^5^ to 10^7^ per sample. The colonies continued to grow until confluency was reached. No CFU growth occurred in plates extracted from group 1 for the entire duration of the incubation period (7 days; Figure 8). Most common bacteria identified included *Staphylococcus* and *Micrococcus*. The common species among them were *Staphylococcus epidermis*, *Staphylococcus aureus*, *Micrococcus luteus*, and *Staphylococcus pettenkoferi*. All these bacteria were only present in group 2 but none in group 1.

**Figure 7.**
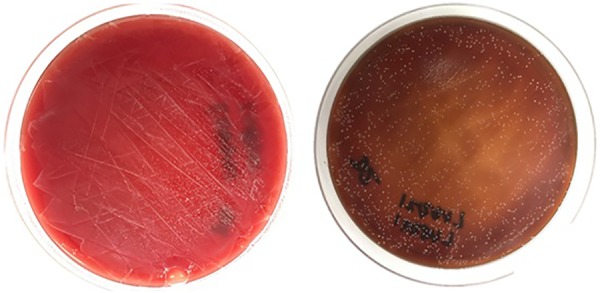
Colony-forming units on sample 2 of group 2, at 24 hours (left) and 4 days (right) after streaking. These represent 2 of 0.1 mL triplicates that were cultured on plates.

**Figure 8.**
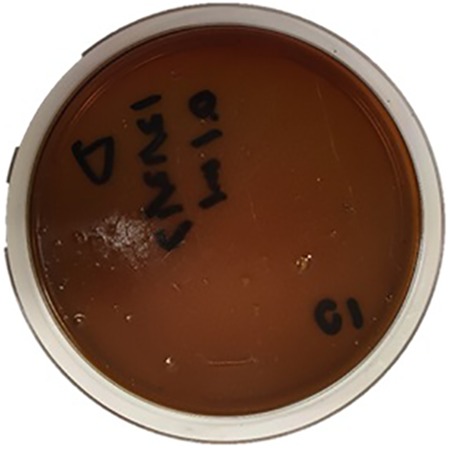
Culture plate representative of samples from group 1 after 7 days. No colony-forming units were found in any 0.1 mL triplicates, extracted from 5 samples. This represents 5 (samples) × 3 (triplicates) = 15 plates. No bacterial species/DNA present.

## Discussion

Recent studies looking at implant cleanliness to reduce risk of infection or inflammatory responses are categorized into (1) preoperative preventative measure and (2) intraoperative preventative measure. Preoperative preventative measure focuses on providing the cleanest and sterile implant, free of contaminants (microbial and nonmicrobial) to the OR. Whereas intraoperative preventative measure focuses on avoiding contamination of the clean and sterile implant, provided to the OR, during the surgery. An example of preoperative preventative measure is the study done by Litrico et al,^8^ where they highlighted evidences of reduced SSI by avoiding repeated reprocessing in the hospital. An example of intraoperative preventative measures is the study done by Rehman et al,^12^ where they highlighted evidence of reducing SSI by using a fresh pair of gloves, every time before touching the implant during the procedure. In the current study, both control (unguarded) and the treatment (guarded) groups are single-use, presterilized pedicle screws, and therefore, the preoperative preventative measure was in place for both the groups. The current study focusses on the second step toward infection or inflammatory risk prevention, that is, the intraoperative preventative measure.

The study presents a pivotal single-center data on a stand-alone method of preventing microbial dose delivery through pedicle screws. The binary nature of the data across all the sample pairs highlights its clinical significance, that is, in terms of dosage, presence and absence of guard is equivalent to presence or absence of microbial contaminants. Four key factors that define the pathogenesis of SSI are the virulence, host site, immunity, and dosage.^16^ The virulence is the microorganism’s ability to infect the host. Although many bacterial species have been identified to cause SSI, the most common ones, *Staphylococcus epidermidis* and *Staphylococcus aureus*, are always present at the vicinity as part of a patient’s own flora. In addition, they have the potential to form biofilms, secluding itself from macrophages or other immune responses at the host site.^17-19^ The host sites in spine surgery are the pedicles of the vertebrae. This in combination with availability of metal surface (pedicle screws) provides a conducive environment for the bacteria to grow. Besides clinical signs of infection, evidences have also shown occurrence of occult infection, which manifests as hardware loosening or dislodgement, thus requiring a revision surgery.^20,21^ Last, the dose dictates how much bacterial bioburden the “sterile” implant carries, after handing and at implantation. This study characterizes the dose and the virulence, that is, type and quantity of bacteria species present, carried through a pedicle screw during spine surgery, alongside establishing an efficient method to mitigate it.

Aseptic surgical techniques rely on methods that reduce or at least avoid transfer of contamination, a majority of which are microbial organisms and not visible to naked eyes. Over the past decade, the use of antibiotics, both local and systematic, have increased enormously. As a by-product this has led to the increase in the number and type of resistant bacterial species, to an extent that the World Health Organization ranked this concern next to issues like terrorism and global warming.^22^ Therefore, any practice that could cost-efficiently reduce the current bioburden beginning transferred to the patients would be desirable. Such a practice has been common in breast augmentation surgeries, where the surgeons use a sterile guard called Keller funnel to handle the breast implants.^14^ Similarly, wound protectors have become the standard of practice in general surgery to avoid contamination of the wound irrigation fluid from the wound edges.^15^ The premise of both these practices includes provision of an impermeable guard. This guard, in essence, avoids contact between the contaminated object and the liquid or implant being delivered into the surgical site. Rehman et al have recently shown a significant reduction in SSI rate by avoiding handling the screw with blood-stained gloves.^12^ In theory, adoption of this practice would require universal education and is dependent on the consistency and compliance of every individual surgeon. Even then, a pedicle screw is held by a scrub tech during unwrapping and attachment to an insertion device, followed by its placement next to other dirty surgical instruments. In contrast, the current study evaluates a guard that provides uninterrupted protection from all the aforementioned elements. Therefore, based on literature, contemporary practices, and the data from the current study, it seems rational to hypothesize that such a guard could mitigate undue infections, and thus reduce the rate of SSI, post spinal fusion.

## Conclusion

The results of this study provide preliminary clinical evidence that further improvement in asepsis is possible through simple cost-efficient innovations in intraoperative practices. The method proposed involves the use of an intraoperative guard, which shields the pedicle screws, until implantation, without affecting other preparatory process like unwrapping, attaching to the instrument, and so on.
